# Associations of the Monocyte to High‐Density Lipoprotein Cholesterol Ratio With Stroke Prevalence and All‐Cause Mortality: Evidence From a Population‐Based Study

**DOI:** 10.1002/brb3.70896

**Published:** 2025-09-26

**Authors:** Qing Meng, Li Zhang, Shengqiang Fan, Bin Shen, Chaoping Zou, Dezhou Sun, Xianghui Liu, Jian Zhang, Shugang Xu

**Affiliations:** ^1^ School of Clinical Medicine Shandong Second Medical University Weifang Shandong Province PR China; ^2^ Neurosurgery Department Dezhou People's Hospital Dezhou Shandong Province PR China

**Keywords:** MHR, monocyte to high‐density lipoprotein cholesterol ratio, mortality, NHANES, stroke

## Abstract

**Background:**

Monocyte to high‐density lipoprotein cholesterol ratio (MHR) is a biomarker of inflammation and metabolic disorders. However, the correlation between MHR and stroke is not well‐studied. This study aims to examine how MHR correlates with stroke prevalence and prognosis.

**Methods:**

We used cross‐sectional and longitudinal methods to analyze data from the National Health and Nutrition Examination Survey (NHANES) spanning 2009–2018. The subjects were divided into four groups based on the MHR quartiles. We evaluated the correlation between MHR and the incidence of stroke through weighted multivariate logistic regression and curve fitting. In order to test whether there were differences between the different subgroups, stratified analyses were constructed, and the capacity of MHR to predict stroke was assessed using receiver operating characteristic (ROC) curves. Furthermore, we explored the connection between MHR and all‐cause mortality by performing Cox proportional hazards models and Kaplan–Meier survival curves in stroke individuals.

**Results:**

We had a total of 17,161 adult participants with a mean age of 49.07 ± 17.58 years of which 593 were diagnosed with stroke, with a prevalence of 3.46%. MHR was found to have a positive correlation with the incidence of stroke when fully adjusting for potential confounding variables. For each unit increase in MHR, the probability of stroke is elevated by 61% (OR: 1.61; 95% CI, 1.17–2.44, *p* = 0.022). Curve fitting analysis revealed a linear relationship between baseline MHR index and stroke (nonlinearity *p* = 0.963). Subgroup analyses indicated that most stratified variables did not significantly interact with the relationship between MHR and stroke, but the effect of MHR was stronger in individuals without coronary heart disease. Furthermore, weighted Cox regression analyses revealed that the odds of all‐cause mortality 72% higher (HR: 1.72; 95% CI, 1.16–2.78, *p* = 0.027) for stroke patients in the highest quartile (Q4), compared with the lowest quartile (Q1) of MHR. Kaplan–Meier curves revealed that all‐cause mortality significantly increased as MHR index values rose.

**Conclusions:**

In this cross‐sectional study of US adults, MHR maintains a linear positive relationship with stroke. In addition, MHR can help predict long‐term mortality in individuals with stroke. The analyses demonstrate that MHR may serve as an effective predictor of stroke and its mortality.

## Graphical abstract

The relationship between the monocyte to high‐density lipoprotein cholesterol ratio (MHR) and stroke remains unclear. This study aimed to explore the association between MHR and stroke prevalence as well as prognosis. The results indicate that the risk of stroke increases progressively with higher MHR levels, and MHR demonstrates a strong predictive ability for long‐term mortality in individuals with stroke.

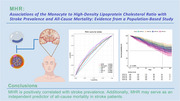

## 1 Introduction

Stroke is a severe acute cerebrovascular event and the second most prevalent cause of death worldwide, resulting in considerable disability and imposing a substantial burden on individuals, families, and society. Over 13.7 million new stroke cases are reported annually, with ischemic stroke making up the majority (Boulanger et al. 2018). In the context of the stroke epidemic in China, previous studies have provided significant epidemiological insights. For adults aged 40 years and older, data from 2020 reveal a stroke prevalence of 2.6%, an incidence rate of 505.2 per 100,000 person‐years, and a mortality rate of 343.4 per 100,000 person‐years. These figures correspond to approximately 17.8 million stroke cases, 3.4 million new stroke events, and 2.3 million deaths related to stroke in China (Tu et al. 2023). Major risk factors for stroke include hypertension, diabetes, obesity, oxidative stress, and inflammation (Boulanger et al. 2018). Despite medical advances have reduced stroke mortality, the incidence continues to rise, particularly in low‐income regions, and early detection and treatment of stroke is particularly important (GBD 2019 Stroke Collaborators 2021). Effective prevention and timely intervention can significantly lower stroke incidence, enhance patient outcomes, and improve quality of life. As such, the early identification of modifiable risk factors, encouragement of healthy lifestyles, and the implementation of comprehensive prevention strategies are essential to alleviating the global burden of stroke (Dirnagl and Endres 2014).

Atherosclerosis‐induced endothelial damage, plaque formation, thrombosis, and stenosis or occlusion of blood vessels are significant risk factors for stroke (Becker and Stroke 2016). Oxidation of low‐density lipoprotein cholesterol (LDL‐C) and monocyte aggregation are central mechanisms in atherosclerosis. When the arterial endothelium is damaged, oxidized‐LDL‐C activates chemokines (MCP‐1) in endothelial cells and promotes monocyte recruitment, leading to lipid accumulation and foam cell formation (Kim and Cho 2016; Zhao et al. 2020), thereby exacerbating the inflammatory response and the atherosclerotic process of the blood vessels. In the early stages of the immune response, monocytes release cytokines such as TNF‐α, IL‐6, and IL‐1β to attract other immune cells (Tirandi et al. 2023; Boutin et al. 2001; Loddick and Rothwell 1996), triggering a localized immune response. Additionally, monocytes can activate neuroimmune interactions, inducing an inflammatory response in neural tissue, which may ultimately destabilize the vasculature (Liberale et al. 2020; Anrather and Iadecola 2016; Low et al. 2019). In contrast, research has shown that high‐density lipoprotein cholesterol (HDL‐C) plays a key role in anti‐inflammatory, antioxidant, and antithrombotic effects. HDL‐C reduces atherosclerotic plaque formation by promoting lipid clearance and cholesterol metabolism. The antioxidant enzymes in HDL, such as glutathione peroxidase and catalase, can scavenge free radicals in the body, inhibiting the oxidation of LDL‐C and preventing ox‐LDL‐C damage to the vascular endothelium (Okamura et al. 2006; Ren et al. 2017).

Monocyte to HDL‐C ratio (MHR) is an emerging biomarker that not only reflects the balance between inflammation and oxidative stress but also demonstrates certain advantages in predicting cardiovascular events over traditional risk factors, such as lipid levels and blood pressure (BP; Zhang et al. 2020; Sercelik et al. 2018). Furthermore, MHR exhibits greater sensitivity in predictive capability than either monocyte count or HDL‐C concentration. However, research exploring the correlation between MHR and stroke is still limited. Therefore, the purpose of our study was to explore the association between MHR and stroke prevalence, as well as its relationship with all‐cause mortality among patients diagnosed with stroke.

## 2 Methods

### 2.1 Study Population and Data Collection

**FIGURE 1:**
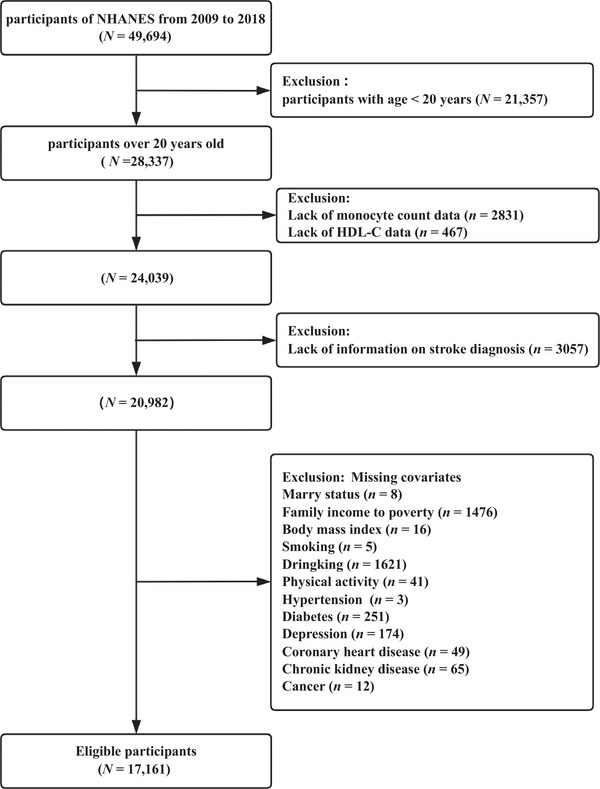
Flow chart of sample selection from the NHANES 2009–2018.

The National Health and Nutrition Examination Survey (NHANES) uses a multistage, stratified, and clustered sampling design to collect nationally representative health examination data on the US population, including basic information on participants, laboratory test results, and underlying diseases. The Research Ethics Review Board of the National Center for Health Statistics (NCHS) has approved the NHANES protocol, and informed consent was provided from every participant. For this research, we utilized data from five cycles of NHANES (2009–2018), which included a cohort of 49,694 participants. Our study excluded the following: individuals under 20 years of age (*n* = 21,357); those with missing data on HDL‐C or monocyte levels (*n* = 4298); those lacking the diagnostic criteria for stroke (*n* = 3057); and those with missing data on key covariates (*n* = 3821). After implementing these exclusions, 17,161 participants were eligible for inclusion in the final analysis (Figure 1).

Sample collection followed strict standardized procedures conducted in Mobile Examination Centers (MECs). Following the collection of venous blood, samples were transported to a central laboratory for analysis. If testing could not be conducted within the designated timeframe, samples were either refrigerated or frozen at the appropriate temperatures. All experimental procedures were meticulously carried out by trained professionals who followed the technical standards (Ahluwalia et al. 2016).

### 2.2 Assessment of MHR

The exposure variable MHR in this study was calculated as monocyte count (10^3^ cells/uL) divided by HDL‐C concentration (mmol/L; Wang et al. 2021). The blood samples of participants were processed using a Beckman Coulter system for complete blood counts, and HDL‐C concentrations were determined by precipitation or immunoassay. All participants were classified into four groups (Q1, Q2, Q3, and Q4) according to the MHR quartiles, with Q1 as the reference group.

### 2.3 Diagnosis of Stroke

Stroke diagnosis was based on self‐reported information from standardized medical questionnaires administered in personal interviews. Participants were inquired: Have you ever been told by a physician or health professional that you have stroke? A positive response was classified as an indication of stroke.

### 2.4 Ascertainment of Outcomes

Follow‐up and mortality data were acquired through the NHANES‐linked NDI public access database, extending until the end of December 2019. The reasons for mortality were classified according to the International Classification of Diseases, 10th Revision (ICD‐10). All‐cause mortality represents the aggregate of deaths due to all specific causes.

### 2.5 Assessment of Covariates

The selection of covariates was guided by relevant studies and clinical expertise, considering multiple potential confounding factors that may affect MHR and stroke. Demographic variables included age, sex, ethnic background, length of schooling, marital status, family income‐to‐poverty ratio (PIR), and body mass index (BMI; < 18.5 kg/m^2^, 18.5–25 kg/m^2^, 25–30 kg/m^2^, ≥ 30 kg/m^2^). Lifestyle factors included smoking behavior (never smoked, former smoker, current smoker), drinking habits (yes/no), and physical activity (PA). Never smokers were defined as people who reported having smoked less than 100 cigarettes in their lifetime; former smokers had smoked more than 100 cigarettes but had quit; the individuals who had smoked more than 100 cigarettes in their lifetime and were still smoking were categorized as current smokers. Alcohol consumers are defined as individuals who drink at least 12 alcoholic beverages annually. The assessment of PA relied on the total metabolic equivalent (MET) minutes each week according to the US Physical Activity Guidelines, with PA categorized as inactive (0 MET‐min/week), insufficiently active (< 600 MET‐min/week), and active (> 600 MET‐min/week). Blood samples were collected to measure neutrophils, lymphocytes, monocytes, and hemoglobin counts. Laboratory measures included albumin, HbA1c, total cholesterol (TC), triglycerides (TG), HDL‐C, and serum creatinine (SCR). Comorbidities include coronary heart disease (CHD), chronic kidney disease (CKD), diabetes, hypertension, hyperlipidemia, and cancer. CKD is defined as self‐reported CKD, eGFR < 60 mL/min/1.73 m^2^, or urine albumin‐to‐creatinine ratio (UACR) ≥ 30 mg/g. The diagnosis of hypertension was determined using one of the subsequent criteria: a physician's diagnosis of hypertension, an average BP ≥ 130/90 mm Hg, or the use of antihypertensive medication. Diabetes is defined as self‐reported diagnosis of diabetes confirmed by a doctor, fasting blood glucose (FPG) levels ≥ 7.0 mmol/L, hemoglobin A1c (HbA1c) ≥ 6.5%, or use of antidiabetic medication or insulin. Depression is indicated by a score of 10 or more on the Patient Health Questionnaire‐9 (PHQ‐9), which measures symptoms of depression. Medication use includes self‐reported use of antidiabetic, antihyperlipidemic, or antihypertensive medications within the last 30 days.

### 2.6 Statistical Analysis

R version 4.20 and the Free Statistics software were utilized for statistical analyses in this study. The data were weighted following NHANES guidelines. Since the study includes data from five cycles (2009–2018), the weight was calculated as WTMEC2YR/5. Continuous variables were expressed as means ± standard deviations (SD), and categorical variables were presented as percentages. To assess differences among participants categorized by MHR quartile, the chi‐square tests for categorical variables, the Kruskal–Wallis test was employed for non‐normally distributed continuous variables, and the *t*‐test for normally distributed continuous variables. Weighted logistic regression or Cox proportional hazards models were performed with appropriate adjustments, and odds ratios (ORs), hazard ratios (HRs), and 95% confidence intervals (CIs) used to evaluate the connection between MHR and stroke, as well as its all‐cause mortality. Model 0 represents a single‐factor regression analysis without any adjustments. Model 1 adjusted for basic demographic factors, including sex, age, education, race, marital status, and PIR. Model 2 included additional adjustments for BMI, smoking behavior, drinking habits, lymphocyte count, PA, neutrophil count, and hemoglobin. Model 3 further adjusted for comorbid conditions and medication use, including hypertension, diabetes, hyperlipidemia, CHD, CKD, depression, cancer, and the use of antihypertensive, hypoglycemic, and lipid‐lowering drugs. Restricted cubic spline (RCS) curves were developed using the covariates from Model 3 to evaluate the nonlinear associations of MHR with both stroke and all‐cause mortality. Kaplan–Meier survival analyses were applied to examine survival probabilities over time and to compare survival differences among the four groups. Stratified analysis of MHR was performed by age, sex, race, BMI, hypertension (yes or no), diabetes (yes or no), hyperlipidemia (yes or no), CHD (yes or no), CKD (yes or no), smoking status (yes or no), and drinking habits. To confirm the robustness of the study findings, two sensitivity analyses were conducted. The first analysis excluded participants who took statins to mitigate potential bias on the study endpoint. The second analysis focused on individuals without CHD. A two‐tailed *p* value less than 0.05 was deemed statistically significant.

## 3 Results

### 3.1 The Baseline Characteristics of Participants

Our study included 17,161 participants, comprising 8450 males and 8711 females, with the mean age of 49.07 ± 17.58 years of which 593 were diagnosed with stroke, with a prevalence of 3.46%. The participants were categorized into four groups according to MHR quartiles: Q1 ≤ 0.29, 0.29 < Q2 ≤ 0.41, 0.41 < Q3 ≤ 0.55, and 0.55 < Q4. Tables 1 and 2 describe the baseline characteristics of subjects on the basis of disease status and MHR quartile, respectively. Participants with a history of stroke were generally older and included a higher percentage of females. In terms of lifestyle, the stroke group had a higher prevalence of smoking and less frequent PA. Additionally, the stroke group exhibited more than double the prevalence of depression, diabetes, CKD, and CHD compared to the nonstroke group. Stroke patients also had higher BMI, and more use lipid‐lowering, antihypertensive, and glucose‐lowering medications. As the MHR quartiles increased, the proportion of individuals with stroke (from 2.75% to 4.60%) rose significantly.

**TABLE 1:** Baseline characteristics of the study population divided by stroke status.

Variables	Total	None stroke	Stroke	*p* value
Participants	17,161	16,568	593	
Sex, *n* (%)				0.459
Male	8450 (49.24)	8165 (49.28)	285 (48.06)	
Female	8711 (50.76)	8403 (50.72)	308 (51.94)	
Age, mean ± SD	49.07 1.94))r	48.49 1.94))r	65.17 1.94))r	< 0.001
Race, *n* (%)				< 0.001
Mexican American	2479 (14.45)	2425 (14.64)	54 (9.11)	
Other Hispanic	1747 (10.18)	1701 (10.27)	46 (7.76)	
Non‐Hispanic White	7457 (43.45)	7157 (43.2)	300 (50.59)	
Non‐Hispanic Black	3455 (20.13)	3303 (19.94)	152 (25.63)	
Other race	2023 (11.79)	1982 (11.96)	41 (6.91)	
Education, *n* (%)				< 0.001
Under high school	3837 (22.35)	3647 (22.01)	190 (32.04)	
High school or equivalent	3809 (22.20)	3650 (22.03)	159 (26.81)	
Above high school	9515 (55.45)	9271 (55.96)	244 (41.15)	
Marriage, *n* (%)				< 0.001
Married or living with a partner	3227 (18.80)	3176 (19.17)	51 (8.60)	
Never married	10,183 (59.34)	9877 (59.61)	306 (51.60)	
Other	3751 (21.86)	3515 (21.22)	236 (39.80)	
PIR, *n* (%)				< 0.001
< 1	3764 (21.93)	3613 (21.81)	151 (25.46)	
1–3	7156 (41.70)	6852 (41.36)	304 (51.26)	
> 3	6241 (36.37)	6103 (36.84)	138 (23.27)	
BMI (kg/m^2^), *n* (%)				0.022
Underweight (< 18.5)	265 (1.54)	254 (1.53)	11 (1.85)	
Normal (18.5–25)	4635 (27.01)	4498 (27.15)	137 (23.10)	
Overweight (25–30)	5615 (32.72)	5433 (32.79)	182 (30.69)	
Obesity (9 30)	6646 (38.73)	6383 (38.53)	263 (44.35)	
Smoking status, *n* (%)				< 0.001
Never smoker	9525 (55.50)	9293 (56.09)	232 (39.12)	
Former smoker	4140 (24.12)	3929 (23.71)	211 (35.58)	
Current smoker	3496 (20.37)	3346 (20.20)	150 (25.30)	
Drinking status, *n* (%)				< 0.001
Nondrinker	4691 (27.34)	4487 (27.08)	204 (34.40)	
Drinker	12,470 (72.66)	12,081 (72.92)	389 (65.60)	
Physical activity, *n* (%)				< 0.001
Inactive	10,212 (59.51)	9802 (59.16)	410 (69.14)	
Insufficiently active	1325 (7.72)	1279 (7.72)	46 (7.76)	
Sufficiently active	5624 (32.77)	5487 (33.12)	137 (23.1)	
Diabetes, *n* (%)	3287 (19.15)	3051 (18.42)	236 (39.8)	< 0.001
Hypertension, *n* (%)	6226 (36.28)	5776 (34.86)	450 (75.89)	< 0.001
Hyperlipidemia, *n* (%)	10,913 (63.59)	10,437 (62.99)	476 (80.27)	< 0.001
Total cholesterol (mmol/L), mean ± SD	4.97 chole	4.98 chole	4.73 chole	< 0.001
Triglycerides (mmol/L), mean ± SD	1.73 ycerid	1.73 ycerid	1.72 ycerid	0.481
HDL‐C (mmol/L), mean ± SD	1.37 /L), d	1.37 /L), d	1.34 /L), d	0.044
MHR, mean ± SD	0.45 /L), d	0.45 /L), d	0.49 /L), d	< 0.001
Monocyte (1000 cells/uL), mean ± SD	0.55 /uL),	0.55 /uL),	0.59 /uL),	< 0.001
Neutrophils (1000 cells/uL), mean ± SD	4.27 ± 1.76	4.26 ± 1.76	4.52 ± 1.76	< 0.001
Lymphocyte (1000 cells/uL), mean ± SD	2.13 /uL),	2.14 /uL),	2.01 /uL),	< 0.001
Hemoglobin (g/dL), mean ±an	14.01 in, (	14.03 in, (	13.60 in, (	< 0.001
Depression, *n* (%)	1570 (9.15)	1458 (8.8)	112 (18.89)	< 0.001
eGFR, mean ±an	94.60 1.22.64	95.34 1.22.23	73.89 1.24.11	< 0.001
UACR, mean ±an	43.91 1.89) 10	41.98 1.89) 10	99.24 1.89) 10	< 0.001
CKD, *n* (%)	2893 (16.86)	2636 (15.91)	257 (43.34)	< 0.001
CHD, *n* (%)	672 (3.92)	572 (3.45)	100 (16.86)	< 0.001
Cancer, *n* (%)	1615 (9.41)	1476 (8.91)	139 (23.44)	< 0.001
All‐cause mortality, *n* (%)	1434 (8.36)	1261 (7.61)	173 (29.17)	< 0.001
Antidiabetic drug use, *n* (%)	1711 (9.97)	1576 (9.51)	135 (22.77)	< 0.001
Antihypertensive drug use, *n* (%)	4731 (27.57)	4333 (26.15)	398 (67.12)	< 0.001
Antihyperlipidemic drug use, *n* (%)	3348 (19.51)	3066 (18.51)	282 (47.55)	< 0.001

Abbreviations: BMI, body mass index; CHD, coronary heart disease; CKD, chronic kidney disease; eGFR, estimated glomerular filtration rate; MHR, monocyte to high‐density lipoprotein cholesterol ratio; PIR, the ratio of family income to poverty; UACR, urine albumin‐to‐creatinine ratio.

**TABLE 2:** Baseline characteristics of the study population divided by MHR quartile.

Variables	Total	Q1 (MHR ≤ 0.29)	Q2 (0.29< MHR ≤ 0.41)	Q3 (0.41< MHR ≤ 0.55)	Q4 (MHR > 0.55)	*p* value
Participants	17,161	4290	4288	4283	4300	
Sex, *n* (%)						< 0.001
Male	8450 (49.24)	1318 (30.72)	1881 (43.87)	2331 (54.42)	2920 (67.91)	
Female	8711 (50.76)	2972 (69.28)	2407 (56.13)	1952 (45.58)	1380 (32.09)	
Age, mean ± SD	49.07 32.09)r	49.59 32.09)r	48.61 32.09)r	48.92 32.09)r	49.15 32.09)r	0.037
Race, *n* (%)						< 0.001
Mexican American	2479 (14.45)	504 (11.75)	628 (14.65)	669 (15.62)	678 (15.77)	
Other Hispanic	1747 (10.18)	397 (9.25)	400 (9.33)	468 (10.93)	482 (11.21)	
Non‐Hispanic White	7457 (43.45)	1639 (38.21)	1783 (41.58)	1920 (44.83)	2115 (49.19)	
Non‐Hispanic Black	3455 (20.13)	1119 (26.08)	965 (22.5)	762 (17.79)	609 (14.16)	
Other race	2023 (11.79)	631 (14.71)	512 (11.94)	464 (10.83)	416 (9.67)	
Education, *n* (%)						< 0.001
Under high school	3837 (22.35)	786 (18.33)	982 (22.90)	990 (23.11)	1079 (25.09)	
High school or equivalent	3809 (22.20)	832 (19.39)	903 (21.06)	969 (22.62)	1105 (25.7)	
Above high school	9515 (55.45)	2672 (62.28)	2403 (56.04)	2324 (54.26)	2116 (49.21)	
Marriage, *n* (%)						< 0.001
Married or living with a partner	3227 (18.80)	844 (19.67)	894 (20.85)	765 (17.86)	724 (16.84)	
Never married	10,183 (59.34)	2449 (57.09)	2466 (57.51)	2593 (60.54)	2675 (62.21)	
Other	3751 (21.86)	997 (23.24)	928 (21.64)	925 (21.60)	901 (20.95)	
PIR, *n* (%)						< 0.001
< 1	3764 (21.93)	800 (18.65)	953 (22.22)	1002 (23.39)	1009 (23.47)	
1–3	7156 (41.70)	1665 (38.81)	1712 (39.93)	1809 (42.24)	1970 (45.81)	
> 3	6241 (36.37)	1825 (42.54)	1623 (37.85)	1472 (34.37)	1321 (30.72)	
BMI (kg/m^2^), *n* (%)						< 0.001
Underweight (< 18.5)	265 (1.54)	132 (3.08)	61 (1.42)	49 (1.14)	23 (0.53)	
Normal (18.5–25)	4635 (27.01)	1769 (41.24)	1243 (28.99)	962 (22.46)	661 (15.37)	
Overweight (25–30)	5615 (32.72)	1334 (31.1)	1408 (32.84)	1454 (33.95)	1419 (33.01)	
Obesity (0 30)	6646 (38.73)	1055 (24.59)	1576 (36.75)	1818 (42.45)	2197 (51.09)	
Smoking status, *n* (%)						< 0.001
Never smoker	9525 (55.50)	2715 (63.29)	2515 (58.65)	2305 (53.82)	1990 (46.28)	
Former smoker	4140 (24.12)	943 (21.98)	1012 (23.60)	1083 (25.29)	1102 (25.63)	
Current smoker	3496 (20.37)	632 (14.73)	761 (17.75)	895 (20.89)	1208 (28.09)	
Drinking status, *n* (%)						< 0.001
Nondrinker	4691 (27.34)	1247 (29.07)	1194 (27.85)	1178 (27.50)	1072 (24.93)	
Drinker	12,470 (72.66)	3043 (70.93)	3094 (72.15)	3105 (72.50)	3228 (75.07)	
Physical activity, *n* (%)						< 0.001
Inactive	10,212 (59.51)	2745 (63.99)	2573 (60.00)	2454 (57.30)	2440 (56.74)	
Insufficiently active	1325 (7.72)	323 (7.53)	315 (7.35)	331 (7.73)	356 (8.28)	
Sufficiently active	5624 (32.77)	1222 (28.48)	1400 (32.65)	1498 (34.98)	1504 (34.98)	
Diabetes, *n* (%)	3287 (19.15)	558 (13.01)	718 (16.74)	907 (21.18)	1104 (25.67)	< 0.001
Hypertension, *n* (%)	6226 (36.28)	1346 (31.38)	1457 (33.98)	1602 (37.4)	1821 (42.35)	< 0.001
Hyperlipidemia, *n* (%)	10,913 (63.59)	1879 (43.8)	2412 (56.25)	2958 (69.06)	3664 (85.21)	< 0.001
Total cholesterol (mmol/L), mean ± SD	4.97 /L1.07	5.16 /L1.03	4.99 /L1.05	4.92 /L1.06	4.82 /L1.12	< 0.001
Triglycerides (mmol/L), mean ± SD	1.73 ycerid	1.16 ycerid	1.50 ycerid	1.82 ycerid	2.44 ycerid	< 0.001
HDL‐C (mmol/L), mean ± SD	1.37 /L), d	1.77 /L), d	1.44 /L), d	1.25 /L), d	1.03 /L), d	< 0.001
Monocyte (1000 cells/uL), mean ± SD	0.55 yte (1	0.39 yte (1	0.49 yte (1	0.58 yte (1	0.75 yte (1	< 0.001
Neutrophils (1000 cells/uL), mean ± SD	4.27 ophils	3.49 ophils	3.98 ± 1.43	4.41 ± 1.43	5.18 ± 1.43	< 0.001
Lymphocyte (1000 cells/uL), mean ± SD	2.13 ocyte	1.83 ocyte	2.05 ocyte	2.21 ocyte	2.45 ocyte	< 0.001
Hemoglobin (g/dL), mean ±an	14.01 ine (	13.62 ine (	13.88 ine (	14.12 ine (	14.42 ine (	< 0.001
Depression, *n* (%)	1570 (9.15)	335 (7.81)	368 (8.58)	399 (9.32)	468 (10.88)	< 0.001
eGFR, mean ±an	94.60 1.88) 1	95.42 1.88) 1	95.33 1.88) 1	94.44 1.88) 1	93.21 1.88) 1	< 0.001
UACR, mean ±an	43.91 1.88) 10	35.35 1.88) 10	35.31 1.88) 14	46.99 1.88) 14	58.02 1.88) 14	0.003
CKD, *n* (%)	2893 (16.86)	633 (14.76)	662 (15.44)	749 (17.49)	849 (19.74)	< 0.001
CHD, *n* (%)	672 (3.92)	82 (1.91)	130 (3.03)	184 (4.30)	276 (6.42)	< 0.001
Cancer, *n* (%)	1615 (9.41)	400 (9.32)	372 (8.68)	424 (9.90)	419 (9.74)	0.211
Stroke, *n* (%)	593 (3.46)	118 (2.75)	137 (3.19)	140 (3.27)	198 (4.60)	< 0.001
Antidiabetic drug use, *n* (%)	1711 (9.97)	269 (6.27)	380 (8.86)	480 (11.21)	582 (13.53)	< 0.001
Antihypertensive drug use, *n* (%)	4731 (27.57)	1003 (23.38)	1092 (25.47)	1235 (28.83)	1401 (32.58)	< 0.001
Antihyperlipidemic drug use, *n* (%)	3348 (19.51)	652 (15.2)	808 (18.84)	879 (20.52)	1009 (23.47)	< 0.001

Abbreviations: BMI, body mass index; CHD, coronary heart disease; CKD, chronic kidney disease; eGFR, estimated glomerular filtration rate; MHR, monocyte to high‐density lipoprotein cholesterol ratio; PIR, the ratio of family income to poverty; UACR, urine albumin‐to‐creatinine ratio.

### 3.2 MHR and Stroke

**FIGURE 2:**
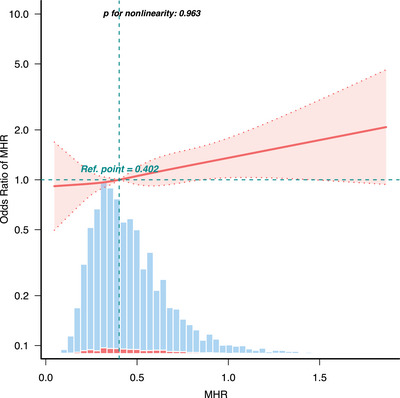
Restricted cubic spline analysis of the association between MHR and the risk of stroke.

**FIGURE 3:**
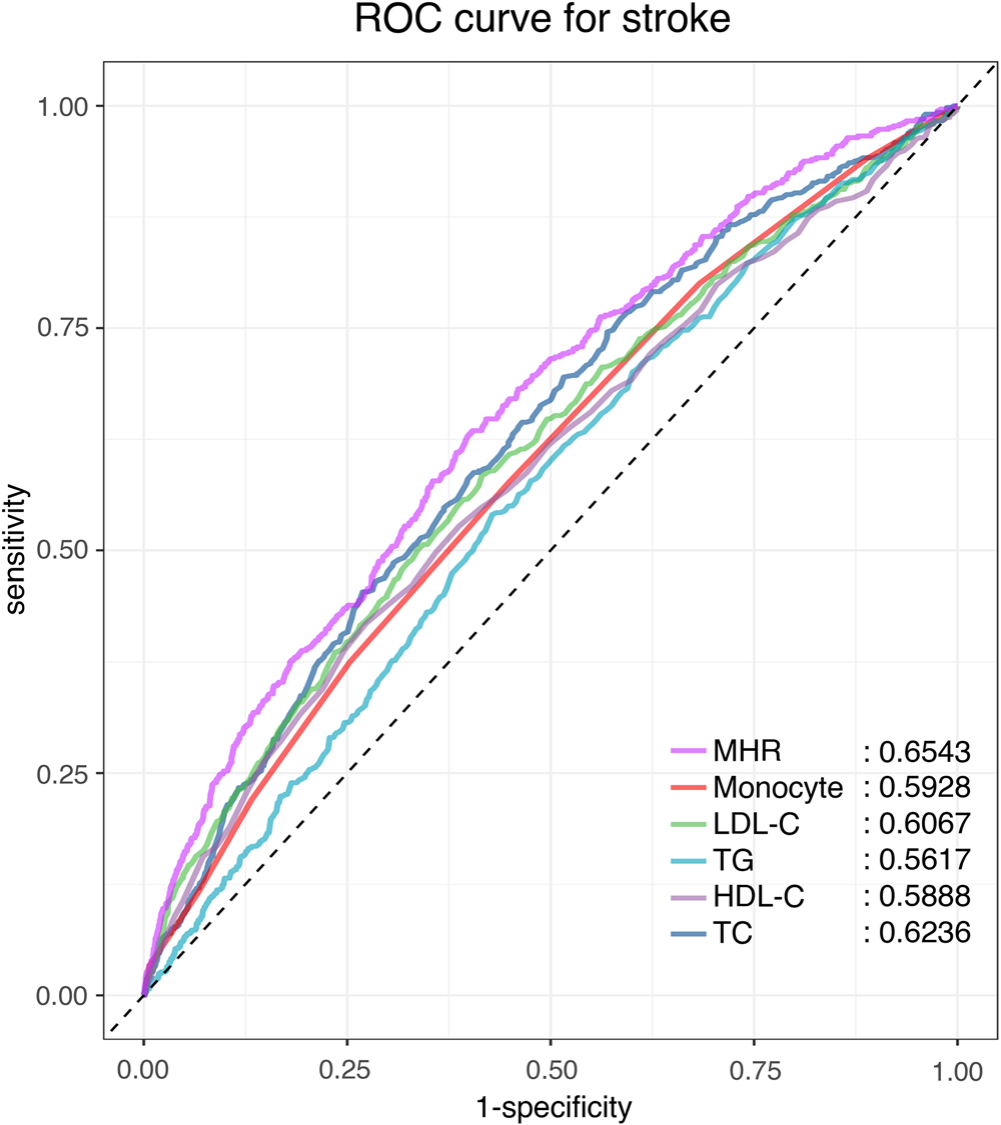
ROC curve analysis of stroke‐related lipid parameters. HDL‐C, high‐density lipoprotein cholesterol; LDL‐C, low‐density lipoprotein cholesterol; MHR, monocyte to high‐density lipoprotein cholesterol ratio; TC, total cholesterol; TG, triglyceride.

As depicted in Table 3, we assessed the relationship between MHR and stroke by using building four different logistic regression analysis models, with the effect size represented by the OR and 95% CI. When MHR was analyzed as a continuous variable and adjusted for multiple potential variables (Model 3), the multivariate logistic regression analysis model produced results that were not significantly different from those of the unadjusted model. In detail, for each unit increase in MHR, the prevalence of stroke rose 61% (OR: 1.61; 95% CI, 1.17–2.44, *p* = 0.022). Furthermore, when MHR was categorized into quartiles, univariate analyses indicated that participants in the highest quartile (Q4) had an elevated risk of stroke compared to those in the lowest quartile (Q1) (Q4: OR: 1.71; 95% CI, 1.35–2.15, *p* < 0.001). After multivariable model adjustment, individuals in Q4 demonstrated a 48% increased risk of stroke (OR: 1.48; 95% CI, 1.12–1.84, *p* = 0.028). Additionally, RCS analysis showed a positive linear correlation between MHR and stroke, with a significant rise in the incidence of stroke as MHR values increased (nonlinear *p* = 0.963; Figure 2). We compared the predictive ability of MHR and other lipid parameters for stroke events. As shown in Figure 3, MHR outperforms traditional lipid markers in predicting stroke. The area under the curve (AUC) for MHR amounted to 0.6543 (95% CI, 0.6307–0.6779), with corresponding sensitivity and specificity values for stroke prediction of 0.627 and 0.5983, respectively (Table S1).

**TABLE 3:** Multivariate logistic analysis of the association between MHR and the risk of stroke.

Variable	Model 0		Model 1		Model 2		Model 3	
	OR (95% CI)	*p* value	OR (95% CI)	*p* value	OR (95% CI)	*p* value	OR (95% CI)	*p* value
MHR continues	2.27 (1.65, 3.13)	< 0.001	2.32 (1.64, 3.29)	< 0.001	2.08 (1.4, 3.08)	< 0.001	1.61 (1.17, 2.44)	0.022
MHR quartiles								
Q1	Reference		Reference		Reference		Reference	
Q2	1.17 (0.91, 1.50)	0.226	1.20 (0.93, 1.55)	0.161	1.19 (0.92, 1.55)	0.183	1.14 (0.87, 1.48)	0.337
Q3	1.19 (0.93, 1.53)	0.161	1.22 (0.94, 1.59)	0.127	1.20 (0.91, 1.57)	0.194	1.06 (0.81, 1.40)	0.659
Q4	1.71 (1.35, 2.15)	< 0.001	1.76 (1.37, 2.26)	< 0.001	1.64 (1.24, 2.16)	0.001	1.48 (1.12, 1.84)	0.028
*p* for trend	< 0.001		< 0.001		0.001		0.039	

Model 0: Nonadjusted.

Model 1: Sex, age, education, race, marital status, and PIR.

Model 2: Model 1 + BMI, smoking status, drinking habits, lymphocyte count, neutrophil count, hemoglobin, and physical activity.

Model 3: Model 2+ hypertension, diabetes, hyperlipidemia, CHD, CKD, depression, cancer, antihypertensive drug, antihyperlipidemic drug, antidiabetic drug.

### 3.3 MHR and Stroke Survivor All‐Cause Mortality

**FIGURE 4:**
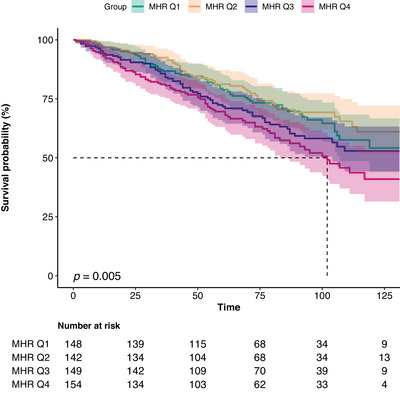
Kaplan–Meier survival analysis of MHR quartiles with all‐cause mortality in stroke patients.

**FIGURE 5:**
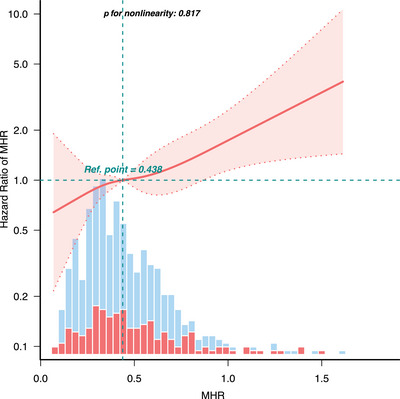
Restricted cubic spline analysis of the association between MHR and all‐cause mortality in stroke patients.

During a median follow‐up of 71.79 months, a total of 173 (29.2%) deaths occurred in the stroke population (Table S2). All‐cause mortality rates among stroke patients in the MHR quartiles were 38 (25.7%), 36 (25.4%), 45 (30.2%), and 54 (35.1%), respectively (Table S3). Kaplan–Meier curves for survival stratified by MHR index suggested differences in all‐cause mortality across different groups (*p* < 0.001), with the lowest survival rate observed in participants in Q4 (Figure 4). RCS models were employed to assess the link between MHR and all‐cause mortality, demonstrating a positive linear correlation (nonlinear *p* = 0.545; Figure 5). Weighted multivariate Cox proportional hazard model analysis also confirmed the correlation between MHR and all‐cause mortality (Table 4). According to the crude model, we found that every unit increase in MHR was associated with a 161% higher risk of all‐cause mortality (HR: 2.61, 95% CI, 1.45–4.68, *p* = 0.001). After controlling for confounding variables (Models 1, 2, and 3), the correlation between MHR and all‐cause mortality remained significant. When MHR was categorized into quartiles, the HRs for MHR Q4 (MHR > 0.58) were 1.59 (95% CI, 1.14–2.46, *p* = 0.032), 1.66 (95% CI, 1.13–2.67, *p* = 0.036), and 1.72 (95% CI, 1.16–2.78, *p* = 0.027) in the crude model and adjusted models (Models 2 and 3), respectively. The findings indicate that MHR serves as a potential predictor of all‐cause mortality in stroke patients.

**TABLE 4:** Multivariate Cox proportional hazard of association between MHR and all‐cause mortality in patients with stroke.

Variable	Model 0		Model 1		Model 2		Model 3	
	HR (95% CI)	*p* value	HR (95% CI)	*p* value	HR (95% CI)	*p* value	HR (95% CI)	*p* value
MHR continues	2.61 (1.45, 4.68)	0.001	2.27 (1.29, 4.34)	0.013	2.67 (1.37, 4.41)	0.004	2.54 (1.42, 4.53)	0.003
MHR quartiles								
Q1	Reference		Reference		Reference		Reference	
Q2	1.06 (0.67, 1.68)	0.791	1.13 (0.71, 1.78)	0.612	1.17 (0.73, 1.89)	0.504	1.09 (0.68, 1.78)	0.694
Q3	1.22 (0.79, 1.90)	0.370	1.36 (0.87, 2.13)	0.178	1.57 (0.98, 2.51)	0.059	1.51 (0.94, 2.42)	0.087
Q4	1.59 (1.14, 2.46)	0.032	1.74 (1.12, 2.68)	0.013	1.66 (1.13, 2.67)	0.036	1.72 (1.16, 2.78)	0.027
*p* for trend	0.022		0.008		0.019		0.014	

Model 0: Nonadjusted.

Model 1: Sex, age, education, race, marital status, and PIR.

Model 2: Model 1 + BMI, smoking status, drinking habits, lymphocyte count, neutrophil count, hemoglobin, and physical activity.

Model 3: Model 2+ hypertension, diabetes, hyperlipidemia, CHD, CKD, depression, cancer, antihypertensive drug, antihyperlipidemic drug, antidiabetic drug.

### 3.4 Stratified Analysis and Sensitivity Analysis

**FIGURE 6:**
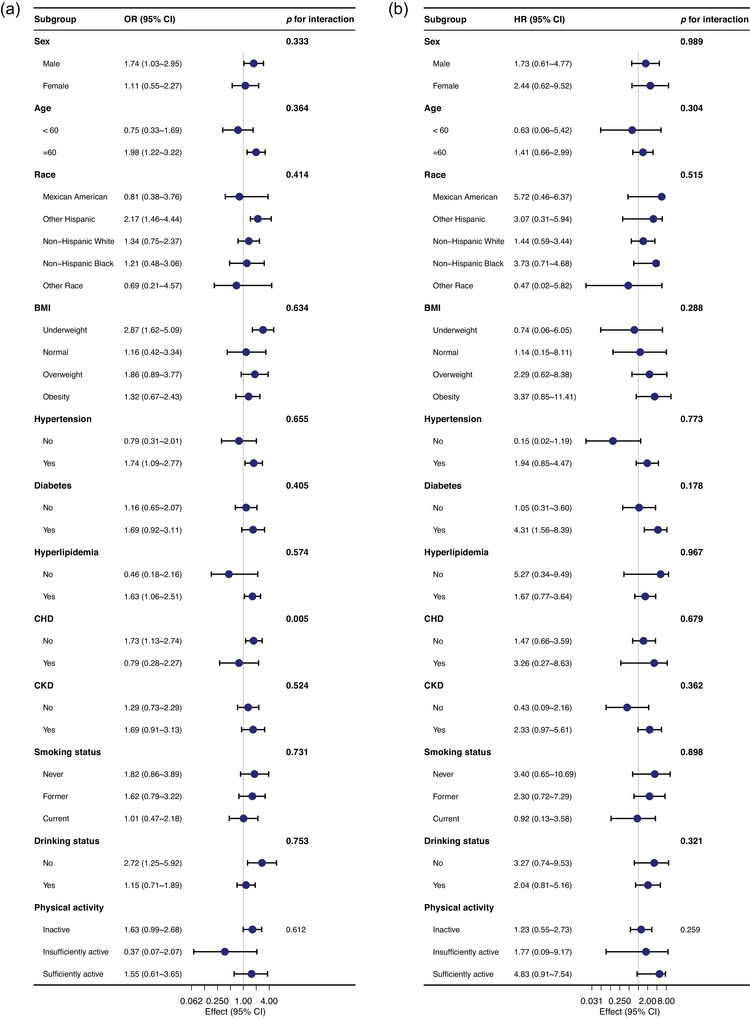
(a) Stratified analysis for the association between MHR and stroke. (b) Stratified analysis for the association between MHR and all‐cause mortality in stroke patients.

Stratified analysis revealed that the association between MHR and stroke, along with all‐cause mortality, remained consistent in the majority of subgroups, including age, sex, race, BMI, hypertension (yes or no), diabetes (yes or no), hyperlipidemia (yes or no), CKD (yes or no), smoking status (never, former, and current), and drinking habits (yes or no). However, a significant interaction between MHR and stroke was found in the non‐CHD population (Figure 6). Sensitivity analysis conducted on the data after removing participants who took statins showed a positive correlation between MHR and stroke, along with all‐cause mortality in stroke survivors (Table S4). Additionally, excluding patients with CHD at baseline resulted in 16,490 individuals remaining in the study cohort. Following adjustment for potential confounders, the relationship between MHR and stroke remained consistent with the main analysis (Table S5).

## 4 Discussion

We conducted a comprehensive cross‐sectional cohort study using the NHANES dataset to examine the connection between MHR and the risk of stroke and mortality. Our findings indicate that, even after adjusting for potential confounders, MHR levels remain independently associated with a heightened stroke risk, with curve fitting analysis suggesting a linear relationship. In stroke patients, MHR is significantly associated with all‐cause mortality. These results are robust whether MHR is considered as a continuous or categorical variable. This study highlights the value of MHR as a dependable indicator for identifying high‐risk individuals for stroke and its clinical potential in prognostic prediction.

MHR, as a biomarker of inflammation, is linked to the development and prognosis of certain cardiovascular diseases (CVDs). For instance, a cohort study involving 34,335 participants from the United States, the general population demonstrated that increased MHR was significantly correlated with susceptibility to CVD mortality, whereas MHR was nonlinearly associated with all‐cause mortality (Jiang et al. 2022). Another observational study identified MHR as a significant independent predictor of ischemic heart failure in patients with both ischemic heart failure and diabetes undergoing percutaneous coronary intervention. It was observed that higher MHR values correlated with an increased occurrence of major adverse cardiovascular events (MACEs; Li et al. 2023). In addition, studies have reported revealing elevated MHR levels as an independent risk factor for chronic renal failure and cardiorenal syndrome (Lin et al. 2024; Xu et al. 2024). The data on the relationship between MHR and stroke are limited. To the best of our knowledge, only two retrospective studies have examined the prognostic role of MHR in long‐term mortality among stroke patients. In a prospective study by Qin and colleagues, higher levels of MHR in patients with ischemic stroke or transient ischemic attack were independently correlated with a heightened risk of adverse outcomes and all‐cause mortality at both 3‐month and 1‐year follow‐ups (Xu et al. 2023). Meanwhile, in another Chinese population‐based follow‐up study of 316 individuals, elevated MHR levels were considered to be independently correlated with an increased risk of 3‐month poor functional outcome in ischemic stroke with large atherosclerosis (Li et al. 2021). One study found that MHR could serve as an independent predictor of emergency large vessel occlusion (ICAS‐ELVO) related to intracranial atherosclerotic stenosis in acute ischemic stroke patients. The research demonstrated that in the training dataset, the monocyte count and MHR were significantly higher in the ICAS group compared to the embolic group. Moreover, in the testing dataset, patients with lower MHR levels experienced more brain hemorrhages, suggesting that MHR may be an important marker for stroke complications. Additionally, another study explored the impact of intravenous tissue plasminogen activator (IV‐tPA) treatment on MHR in acute ischemic stroke patients (Lin et al. 2024). Their study found that elevated MHR levels after IV‐tPA treatment were linked to an increased risk of early neurological deterioration (END) and hemorrhagic transformation (HT). Specifically, the MHR values in patients with END and HT were significantly higher than in those without these complications, indicating that MHR could serve as a predictor for adverse events post‐treatment (Ruorui et al. 2023). Our research further confirmed the positive correlation between MHR and risk of stroke incidence, with stratified analysis revealing a notable interaction between MHR and individuals without CHD. Cox regression and curve fitting analyses demonstrate a linear correlation between MHR and mortality among stroke patients. MHR has the potential to become a valuable tool for identifying high‐risk individuals for stroke, while also holding significant value for the long‐term clinical outcomes of stroke survivors.

Research on the pathophysiological mechanisms of MHR is limited, and MHR may elevate the risk of stroke and mortality through mechanisms such as lipid imbalance, endothelial dysfunction, and inflammatory factors. Atherosclerosis serves as a key physiological foundation for the occurrence of stroke, with monocytes involved in the inflammation, oxidative stress, and immune mechanisms throughout the entire disease process (Lambertsen et al. 2012). In the early stages of atherosclerosis, damaged or activated vascular endothelial cells release ICAM‐1 and VCAM‐1, which promote interactions between monocytes and endothelial cells (Becker and Stroke 2016; Georgakis et al. 2019). Meanwhile, under the influence of chemokine MCP‐1 secreted by endothelial cells, monocytes are guided to migrate into the subendothelial space, where they differentiate into macrophages (Zhao et al. 2020; Winter et al. 2018). Subsequently, during the oxidative stress process mediated by reactive oxygen species (ROS), macrophages recognize and engulf ox‐LDL‐C through surface receptors (CD36, SR‐A1), leading to the formation of foam cells (Libby 2012; Rajamäki et al. 2010), thereby accelerating atherosclerotic plaque formation and progression, which directly increases the risk of stroke. Inflammation is a key factor in atherosclerosis, thrombosis, and cerebral small vessel disease, and it is correlated with the severity and outcomes of stroke. Peripheral immune cells, particularly monocytes, produce proinflammatory cytokines and chemokines that disrupt the blood–brain barrier (BBB; Bai et al. 2023). Matrix metalloproteinases (MMP) lead to the degradation of the BBB and extracellular matrix (ECM), resulting in cerebral edema and cytokine leakage, thereby damaging brain tissue (Mechtouff et al. 2020). Conversely, HDL‐C is considered to have antiatherosclerotic and anti‐inflammatory properties. HDL‐C exerts its reverse cholesterol transport function by regulating various cholesterol transporters (ABCA1, ABCG1, SR‐BI), transferring cholesterol and lipids from foam cells within plaques back to the liver for metabolism, thereby reducing lipid accumulation in the plaques and contributing to plaque stability (Ossoli et al. 2016). Studies have shown that apolipoprotein A‐1 (ApoA‐1) in HDL‐C can inhibit the activation of CD11b, reduce monocyte adhesion and infiltration (Feig et al. 2016; Nagao et al. 2018). It also regulates the transcription factor ATF3 to decrease transmission of the Toll‐like receptor (TLR) signaling pathway and inhibit monocyte function. HDL suppresses the oxidation of LDL by inhibiting paraoxonase 1 (PON1) to reduce oxidative stress in endothelial cells, and triggers the endothelial nitric oxide synthase (eNOS) pathway to generate NO, which promotes endothelial cell regeneration (Yuhanna et al. 2001; Hong et al. 2019). MHR combines two independent risk factors, monocyte count and HDL‐C. Its elevated levels may better reflect endothelial dysfunction and dyslipidemia, and it could serve as an effective surrogate marker for systemic inflammation.

This study may be the first to examine the linear relationship between MHR and the risk of stroke in the adult population in the United States. After adjusting for multiple confounding variables, each one‐unit rise in the baseline MHR value corresponds to a 61% greater risk of stroke and a 154% higher chance of all‐cause mortality. The present study has some strengths. It utilizes national‐level data, offering a large sample size and detailed participant information, which strengthens the representativeness and stability of the findings. Additionally, we adjusted for confounding factors, providing best evidence for the association between MHR and stroke incidence. We applied weighting adjustments according to NHANES analytical guidelines and performed sensitivity analyses, with results consistent with the primary analysis, thereby increasing the study's credibility. However, some limitations should be mentioned. First, the NHANES database includes only baseline information at the time of monocyte and HDL‐c measurement, which may not capture dynamic changes over time. For example, fluctuations in monocyte count and HDL‐C levels due to short‐term health changes or lifestyle modifications are not taken into account. Second, this was a retrospective study, so a causal association between MHR and stroke cannot be established. Additionally, although we have accounted for several potential confounders, there may still be residual confounding. Unmeasured variables, such as genetic factors and environmental exposures, could introduce bias into the results. For example, certain genetic mutations might affect both monocyte function and lipid metabolism, thereby possibly influencing the MHR–stroke relationship. Moreover, the diagnosis of stroke was based on self‐reported medical history. This approach lacks objective indicators from imaging and clinical history, and there is no specific information on clinical subtypes or staging. As a result, the data may be subject to recall bias, and misclassification of stroke cases could have occurred. Therefore, future studies should incorporate imaging data to explore the relationship between MHR and specific stroke subtypes.

## 5 Conclusions

In conclusion, the findings of our study indicate that MHR is positively correlated with stroke prevalence. Additionally, MHR may serve as an independent predictor of all‐cause mortality in stroke patients. Given that MHR is easily accessible and economically feasible, it may serve as a promising biomarker with significant potential for stroke diagnosis and prognostic assessment.

## Author Contributions

**Qing Meng**: conceptualization, methodology, writing – original draft, software, writing – review and editing, visualization. **Li Zhang**: data curation, validation, software, visualization. **Shengqiang Fan**: writing – original draft, writing – review and editing, data curation. **Bin Shen**: software, data curation, validation. **Chaoping Zou**: formal analysis, validation, investigation. **Dezhou Sun**: supervision, funding acquisition, project administration, formal analysis, resources. **Xianghui Liu**: supervision, formal analysis, funding acquisition, project administration, resources. **Jian Zhang**: conceptualization, methodology, writing – original draft. **Shugang Xu**: conceptualization, methodology, writing – review and editing.

## Ethics Statement

The NHANES was approved by the National Center for Health Statistics (NCHS) Ethics Review Committee.

## Consent

All participants have signed informed consent forms.

## Conflicts of Interest

The authors declare no conflicts of interest.

## Peer Review

The peer review history for this article is available at https://publons.com/publon/10.1002/brb3.70896.

## Supporting information




**Supporting Information**: brb370896‐sup‐0001‐SuppMat.docx

## Data Availability

All data analyzed can be accessed publicly on the website (https://www.cdc.gov/nchs/nhanes/).
