# Supplementary material for: Associations of the Monocyte to High‐Density Lipoprotein Cholesterol Ratio With Stroke Prevalence and All‐Cause Mortality: Evidence From a Population‐Based Study
Source: Brain Behav. 2025 Sep 26;15(10):e70896. doi: 10.1002/brb3.70896 (PMC12474853; doi:10.1002/brb3.70896)
Supplement: Supplementary file 1 — Supporting Information: brb370896‐sup‐0001‐SuppMat.docx [file BRB3-15-e70896-s001.docx]

**Table S1.** Comparison of MHR and lipid parameters in predicting stroke.

| **Variable** | **AUC (95%CI)** | **Best threshold** | **Sensitivity** | **Specificity** |
| --- | --- | --- | --- | --- |
| MHR | 0.6543 (0.6307, 0.6779) | 0.4763 | 0.6327 | 0.5983 |
| Monocyte | 0.5928 (0.5688, 0.6168) | 0.5501 | 0.5764 | 0.5521 |
| LDL-C | 0.6067 (0.5814, 0.6321) | 2.8327 | 0.5837 | 0.5855 |
| TG | 0.5617 (0.5376, 0.5857) | 0.8726 | 0.5439 | 0.5706 |
| HDL-C | 0.5888 (0.5631, 0.6146) | 1.0749 | 0.6148 | 0.5632 |
| TC | 0.6236 (0.5997, 0.6475) | 4.5651 | 0.7016 | 0.4503 |

Abbreviations: MHR, monocyte to high-density lipoprotein cholesterol ratio; LDL-C, low-density lipoprotein cholesterol; HDL-C, high-density lipoprotein cholesterol; TC, total cholesterol; TG, triglyceride.

**Table S2.** Baseline characteristics of the study population divided by all-cause mortality among stroke survivors.

| **Variables** | Total | Alive | All-cause death | ***p*-value** |
| --- | --- | --- | --- | --- |
| Participants | 593 | 420 | 173 |  |
| Sex, n (%) |  |  |  | 0.012 |
| Male | 285 (48.06) | 188 (44.76) | 97 (56.07) |  |
| Female | 308 (51.94) | 232 (55.24) | 76 (43.93) |  |
| Age, Mean ± SD | 65.17 ± 12.93 | 62.30 ± 13.08 | 72.13 ± 9.47 | < 0.001 |
| Race, n (%) |  |  |  | < 0.001 |
| Mexican American | 54 ( 9.11) | 39 (9.29) | 15 (8.67) |  |
| Other Hispanic | 46 ( 7.76) | 38 (9.05) | 8 (4.62) |  |
| Non-Hispanic White | 300 (50.59) | 186 (44.29) | 114 (65.9) |  |
| Non-Hispanic Black | 152 (25.63) | 121 (28.81) | 31 (17.92) |  |
| Other Race | 41 ( 6.91) | 36 (8.57) | 5 (2.89) |  |
| Education, n (%) |  |  |  | 0.224 |
| Under high school | 190 (32.04) | 130 (30.95) | 60 (34.68) |  |
| High school or equivalent | 159 (26.81) | 108 (25.71) | 51 (29.48) |  |
| Above high school | 244 (41.15) | 182 (43.33) | 62 (35.84) |  |
| Marriage, n (%) |  |  |  | 0.027 |
| Married or living with a partner | 51 ( 8.60) | 40 (9.52) | 11 (6.36) |  |
| Never married | 306 (51.60) | 227 (54.05) | 79 (45.66) |  |
| Other | 236 (39.80) | 153 (36.43) | 83 (47.98) |  |
| PIR, n (%) |  |  |  | 0.089 |
| <1 | 151 (25.46) | 116 (27.62) | 35 (20.23) |  |
| 1-3 | 304 (51.26) | 204 (48.57) | 100 (57.80) |  |
| >3 | 138 (23.27) | 100 (23.81) | 38 (21.97) |  |
| BMI(kg/m2), n (%) |  |  |  | 0.045 |
| Underweight (<18.5) | 11 ( 1.85) | 5 (1.19) | 6 (3.47) |  |
| Normal (18.5–25) | 137 (23.10) | 89 (21.19) | 48 (27.75) |  |
| Overweight (25–30) | 182 (30.69) | 128 (30.48) | 54 (31.21) |  |
| Obesity (≥30) | 263 (44.35) | 198 (47.14) | 65 (37.57) |  |
| Smoking status, n (%) |  |  |  | 0.001 |
| Never smoker | 232 (39.12) | 175 (41.67) | 57 (32.95) |  |
| Former smoker | 211 (35.58) | 130 (30.95) | 81 (46.82) |  |
| Current smoker | 150 (25.30) | 115 (27.38) | 35 (20.23) |  |
| Drinking status, n (%) |  |  |  | 0.922 |
| Nondrinker | 204 (34.40) | 145 (34.52) | 59 (34.10) |  |
| drinker | 389 (65.60) | 275 (65.48) | 114 (65.90) |  |
| Physical Activity, n (%) |  |  |  | < 0.001 |
| inactive | 410 (69.14) | 267 (63.57) | 143 (82.66) |  |
| insufficiently active | 46 ( 7.76) | 35 (8.33) | 11 (6.36) |  |
| Sufficiently active | 137 (23.10) | 118 (28.1) | 19 (10.98) |  |
| Diabetes, n (%) | 236 (39.80) | 158 (37.62) | 78 (45.09) | 0.019 |
| Hypertension, n (%) | 450 (75.89) | 309 (73.57) | 141 (81.50) | 0.039 |
| Hyperlipidemia, n (%) | 476 (80.27) | 341 (81.19) | 135 (78.03) | 0.376 |
| Total cholesterol (mmol/L), Mean ± SD | 4.73 ± 1.12 | 4.76 ± 1.07 | 4.65 ± 1.25 | 0.289 |
| Triglycerides (mmol/L), Mean ± SD | 1.72 ± 1.11 | 1.72 ± 1.07 | 1.73 ± 1.19 | 0.914 |
| HDL-C (mmol/L), Mean ± SD | 1.34 ± 0.44 | 1.34 ± 0.41 | 1.35 ± 0.49 | 0.796 |
| MHR, Mean ± SD | 0.49 ± 0.26 | 0.47 ± 0.24 | 0.54 ± 0.29 | 0.007 |
| Monocyte (1000cells/uL), Mean ± SD | 0.59 ± 0.21 | 0.57 ± 0.19 | 0.64 ± 0.24 | < 0.001 |
| Neutrophils (1000 cells/uL), Mean ± SD | 4.52 ± 1.72 | 4.39 ± 1.61 | 4.85 ± 1.94 | 0.003 |
| Lymphocyte (1000cells/uL), Mean ± SD | 2.01 ± 0.75 | 2.07 ± 0.71 | 1.85 ± 0.83 | 0.289 |
| Haemoglobin（g/dL), Mean ± SD | 13.60 ± 1.63 | 13.66 ± 1.52 | 13.46 ± 1.87 | 0.163 |
| Depression, n (%) | 112 (18.89) | 87 (20.71) | 25 (14.45) | 0.077 |
| eGFR, Mean ± SD | 73.89 ± 24.11 | 77.74 ± 23.25 | 64.58 ± 23.67 | < 0.001 |
| ACR, Mean ± SD | 99.24 ± 357.81 | 85.36 ± 309.50 | 133.73 ± 455.56 | 0.043 |
| CKD, n (%) | 257 (43.34) | 145 (34.52) | 112 (64.74) | < 0.001 |
| CHD, n (%) | 100 (16.86) | 59 (14.05) | 41 (23.7) | 0.004 |
| Cancer, n (%) | 139 (23.44) | 82 (19.52) | 57 (32.95) | < 0.001 |
| Antidiabetic Drug Use, n (%) | 135 (22.77) | 90 (21.43) | 45 (26.01) | 0.226 |
| Antihypertensive Drug Use, n (%) | 398 (67.12) | 271 (64.52) | 127 (73.41) | 0.036 |
| Antihyperlipidemic Drug Use, n (%) | 282 (47.55) | 208 (49.52) | 74 (42.77) | 0.135 |

Abbreviation: MHR, monocyte to high-density lipoprotein cholesterol ratio; PIR, The ratio of family income to poverty; BMI, Body Mass Index; eGFR: estimated glomerular filtration rate; UACR:urine albumin-to-creatinine ratio; CKD,chronic kidney disease; CHD: coronary heart disease.

**Table S3.** Baseline characteristics of the study population divided by MHR Quartile among stroke survivors.

| **Variables** | Total | Q1(MHR≤ 0.31 ) | Q2(0.31<MHR≤0.43 ) | Q3(0.43<MHR≤0.61 ) | Q4(MHR>0.61 ) | ***p*-value** |
| --- | --- | --- | --- | --- | --- | --- |
| Participants | 593 | 148 | 142 | 149 | 154 |  |
| Sex, n (%) |  |  |  |  |  | < 0.001 |
| Male | 285 (48.06) | 44 (29.73) | 54 (38.03) | 80 (53.69) | 107 (69.48) |  |
| Female | 308 (51.94) | 104 (70.27) | 88 (61.97) | 69 (46.31) | 47 (30.52) |  |
| Age, Mean ± SD | 65.17 ± 12.93 | 64.30 ± 13.16 | 65.75 ± 12.72 | 64.76 ± 13.68 | 65.88 ± 12.18 | 0.668 |
| Race, n (%) |  |  |  |  |  | 0.009 |
| Mexican American | 54 ( 9.11) | 14 (9.46) | 12 (8.45) | 14 (9.40) | 14 (9.09) |  |
| Other Hispanic | 46 ( 7.76) | 8 (5.41) | 12 (8.45) | 13 (8.72) | 13 (8.44) |  |
| Non-Hispanic White | 300 (50.59) | 61 (41.22) | 66 (46.48) | 75 (50.34) | 98 (63.64) |  |
| Non-Hispanic Black | 152 (25.63) | 52 (35.14) | 42 (29.58) | 38 (25.50) | 20 (12.99) |  |
| Other Race | 41 ( 6.91) | 13 (8.78) | 10 (7.04) | 9 (6.04) | 9 (5.84) |  |
| Education, n (%) |  |  |  |  |  | 0.214 |
| Under high school | 190 (32.04) | 38 (25.68) | 42 (29.58) | 57 (38.26) | 53 (34.42) |  |
| High school or equivalent | 159 (26.81) | 40 (27.03) | 38 (26.76) | 35 (23.49) | 46 (29.87) |  |
| Above high school | 244 (41.15) | 70 (47.3) | 62 (43.66) | 57 (38.26) | 55 (35.71) |  |
| Marriage, n (%) |  |  |  |  |  | 0.024 |
| Married or living with a partner | 51 ( 8.60) | 13 (8.78) | 11 (7.75) | 16 (10.74) | 11 (7.14) |  |
| Never married | 306 (51.60) | 59 (39.86) | 76 (53.52) | 79 (53.02) | 92 (59.74) |  |
| Other | 236 (39.80) | 76 (51.35) | 55 (38.73) | 54 (36.24) | 51 (33.12) |  |
| PIR, n (%) |  |  |  |  |  | 0.748 |
| <1 | 151 (25.46) | 36 (24.32) | 38 (26.76) | 38 (25.5) | 39 (25.32) |  |
| 1-3 | 304 (51.26) | 74 (50) | 67 (47.18) | 77 (51.68) | 86 (55.84) |  |
| >3 | 138 (23.27) | 38 (25.68) | 37 (26.06) | 34 (22.82) | 29 (18.83) |  |
| BMI(kg/m2), n (%) |  |  |  |  |  | < 0.001 |
| Underweight (<18.5) | 11 ( 1.85) | 5 (3.38) | 3 (2.11) | 1 (0.67) | 2 (1.3) |  |
| Normal (18.5–25) | 137 (23.10) | 48 (32.43) | 40 (28.17) | 25 (16.78) | 24 (15.58) |  |
| Overweight (25–30) | 182 (30.69) | 49 (33.11) | 35 (24.65) | 46 (30.87) | 52 (33.77) |  |
| Obesity (≥30) | 263 (44.35) | 46 (31.08) | 64 (45.07) | 77 (51.68) | 76 (49.35) |  |
| Smoking status, n (%) |  |  |  |  |  | 0.027 |
| Never smoker | 232 (39.12) | 72 (48.65) | 57 (40.14) | 57 (38.26) | 46 (29.87) |  |
| Former smoker | 211 (35.58) | 42 (28.38) | 55 (38.73) | 56 (37.58) | 58 (37.66) |  |
| Current smoker | 150 (25.30) | 34 (22.97) | 30 (21.13) | 36 (24.16) | 50 (32.47) |  |
| Drinking status, n (%) |  |  |  |  |  | 0.165 |
| Nondrinker | 204 (34.40) | 52 (35.14) | 55 (38.73) | 55 (36.91) | 42 (27.27) |  |
| drinker | 389 (65.60) | 96 (64.86) | 87 (61.27) | 94 (63.09) | 112 (72.73) |  |
| Physical Activity, n (%) |  |  |  |  |  | 0.332 |
| inactive | 410 (69.14) | 110 (74.32) | 91 (64.08) | 98 (65.77) | 111 (72.08) |  |
| insufficiently active | 46 ( 7.76) | 12 (8.11) | 10 (7.04) | 14 (9.40) | 10 (6.49) |  |
| sufficiently active | 137 (23.10) | 26 (17.57) | 41 (28.87) | 37 (24.83) | 33 (21.43) |  |
| Diabetes, n (%) | 236 (39.80) | 43 (29.05) | 49 (34.51) | 66 (44.3) | 78 (50.65) | < 0.001 |
| Hypertension, n (%) | 450 (75.89) | 99 (66.89) | 108 (76.06) | 120 (80.54) | 123 (79.87) | 0.022 |
| Hyperlipidemia, n (%) | 476 (80.27) | 95 (64.19) | 111 (78.17) | 130 (87.25) | 140 (90.91) | < 0.001 |
| Total cholesterol (mmol/L), Mean ± SD | 4.73 ± 1.12 | 4.93 ± 1.05 | 4.92 ± 1.18 | 4.68 ± 1.12 | 4.40 ± 1.07 | < 0.001 |
| Triglycerides (mmol/L), Mean ± SD | 1.72 ± 1.11 | 1.18 ± 0.63 | 1.53 ± 0.92 | 1.91 ± 1.32 | 2.22 ± 1.14 | < 0.001 |
| HDL-C (mmol/L), Mean ± SD | 1.34 ± 0.44 | 1.75 ± 0.46 | 1.43 ± 0.32 | 1.22 ± 0.27 | 0.98 ± 0.22 | < 0.001 |
| Monocyte (1000cells/uL), Mean ± SD | 0.59 ± 0.21 | 0.40 ± 0.11 | 0.53 ± 0.13 | 0.62 ± 0.14 | 0.79 ± 0.21 | < 0.001 |
| Neutrophils (1000 cells/uL), Mean ± SD | 4.52 ± 1.72 | 3.79 ± 1.71 | 4.22 ± 1.42 | 4.62 ± 1.60 | 5.42 ± 1.71 | < 0.001 |
| Lymphocyte (1000cells/uL), Mean ± SD | 2.01 ± 0.75 | 1.72 ± 0.62 | 2.00 ± 0.71 | 2.11 ± 0.76 | 2.19 ± 0.84 | < 0.001 |
| Haemoglobin（g/dL), Mean ± SD | 13.60 ± 1.63 | 13.39 ± 1.40 | 13.49 ± 1.48 | 13.67 ± 1.76 | 13.85 ± 1.80 | 0.041 |
| Depression, n (%) | 112 (18.89) | 31 (20.95) | 29 (20.42) | 26 (17.45) | 26 (16.88) | 0.745 |
| eGFR, Mean ± SD | 73.89 ± 24.11 | 77.93 ± 23.33 | 73.91 ± 23.94 | 72.66 ± 25.75 | 71.18 ± 23.07 | 0.091 |
| ACR, Mean ± SD | 99.24 ± 357.81 | 47.40 ± 156.73 | 66.11 ± 227.14 | 164.36 ± 487.72 | 116.18 ± 430.15 | 0.025 |
| CKD, n (%) | 257 (43.34) | 59 (39.86) | 62 (43.66) | 67 (44.97) | 69 (44.81) | 0.794 |
| CHD, n (%) | 100 (16.86) | 18 (12.16) | 28 (19.72) | 26 (17.45) | 28 (18.18) | < 0.001 |
| Cancer, n (%) | 139 (23.44) | 36 (24.32) | 40 (28.17) | 31 (20.81) | 32 (20.78) | 0.389 |
| all-cause mortality, n (%) | 173 (29.17) | 38 (25.68) | 36 (25.35) | 45 (30.2) | 54 (35.06) | 0.028 |
| Antidiabetic Drug Use, n (%) | 135 (22.77) | 19 (12.84) | 31 (21.83) | 37 (24.83) | 48 (31.17) | 0.002 |
| Antihypertensive Drug Use, n (%) | 398 (67.12) | 81 (54.73) | 99 (69.72) | 105 (70.47) | 113 (73.38) | 0.003 |
| Antihyperlipidemic Drug Use, n (%) | 282 (47.55) | 55 (37.16) | 69 (48.59) | 77 (51.68) | 81 (52.6) | < 0.001 |

Abbreviation: MHR, monocyte to high-density lipoprotein cholesterol ratio; PIR, The ratio of family income to poverty; BMI, Body Mass Index; eGFR: estimated glomerular filtration rate; UACR:urine albumin-to-creatinine ratio; CKD,chronic kidney disease; CHD: coronary heart disease.

**Table S4a.** Association between MHR and the risk of stroke after exclusion of participants with CHD.

| **Variable** | **Model 0** | | **Model 1** | | **Model 2** | | **Model 3** | |
| --- | --- | --- | --- | --- | --- | --- | --- | --- |
|  | **OR (95%CI)** | ***p-*value** | **OR (95%CI)** | ***p*-value** | **OR (95%CI)** | ***p*-value** | **OR (95%CI)** | ***p*-value** |
| MHR continues | 2.29 (1.6, 3.26) | <0.001 | 2.54 (1.74, 3.73) | <0.001 | 2.21 (1.43, 3.43) | <0.001 | 1.85 (1.18, 2.88) | 0.007 |
| MHR quartiles |  |  |  |  |  |  |  |  |
| Q1 | Reference |  | Reference |  | Reference |  | Reference |  |
| Q2 | 1.22 (0.93, 1.61) | 0.144 | 1.28 (0.97, 1.69) | 0.086 | 1.25 (0.94, 1.66) | 0.117 | 1.29 (1.01, 1.68) | 0.207 |
| Q3 | 1.15 (0.88, 1.52) | 0.309 | 1.22 (0.92, 1.63) | 0.163 | 1.16 (0.86, 1.56) | 0.321 | 1.41 (1.11, 1.71) | 0.746 |
| Q4 | 1.68 (1.31, 2.17) | <0.001 | 1.85 (1.40, 2.43) | <0.001 | 1.66 (1.22, 2.25) | 0.001 | 1.43 (1.24, 1.95) | 0.022 |
| *p* for trend | <0.001 | | <0.001 | | 0.003 | | 0.034 | |

Model 0: Non-adjusted.

Model 1: Sex, age, education, race, marital status, and PIR.

Model 2: Model 1 + BMI, smoking status, drinking habits, lymphocyte count, neutrophil count, hemoglobin, and physical activity.

Model 3: Model 2+ hypertension, diabetes, hyperlipidemia, CKD, depression, cancer, anti-hypertensive drug, anti-hyperlipidemic drug, anti-diabetic drug.

**Table S4b.** Association between MHR and all-cause mortality after exclusion of participants with CHD.

| **Variable** | **Model 0** | | **Model 1** | | **Model 2** | | **Model 3** | |
| --- | --- | --- | --- | --- | --- | --- | --- | --- |
|  | **HR (95%CI)** | ***p*-value** | **HR (95%CI)** | ***p*-value** | **HR (95%CI)** | ***p*-value** | **HR (95%CI)** | ***p*-value** |
| MHR continues | 2.64 (1.35, 5.15) | 0.004 | 2.35 (1.13, 4.87) | 0.022 | 2.49 (1.17, 5.26) | 0.017 | 2.64 (1.09, 6.41) | 0.032 |

Model 0: Non-adjusted.

Model 1: Sex, age, education, race, marital status, and PIR.

Model 2: Model 1 + BMI, smoking status, drinking habits, lymphocyte count, neutrophil count, hemoglobin, and physical activity.

Model 3: Model 2+ hypertension, diabetes, hyperlipidemia, CKD, depression, cancer, anti-hypertensive drug, anti-hyperlipidemic drug, anti-diabetic drug.

**Table S5a.** Association between MHR and the risk of stroke after exclusion of participants on anti-hyperlipidemic drug.

| **Variable** | **Model 0** | | **Model 1** | | **Model 2** | | **Model 3** | |
| --- | --- | --- | --- | --- | --- | --- | --- | --- |
|  | **HR (95%CI)** | ***p*-value** | **HR (95%CI)** | ***p*-value** | **HR (95%CI)** | ***p*-value** | **HR (95%CI)** | ***p*-value** |
| MHR continues | 1.77 (1.12, 2.82) | 0.015 | 2.03 (1.25, 3.23) | 0.004 | 1.81 (1.15, 3.11) | 0.033 | 1.82 (1.06, 3.13) | 0.037 |
| MHR quartiles |  |  |  |  |  |  |  |  |
| Q1 | Reference |  | Reference |  | Reference |  | Reference |  |
| Q2 | 1.16 (0.84, 1.61) | 0.366 | 1.28 (0.91, 1.78) | 0.153 | 1.25 (0.89, 1.75) | 0.204 | 1.24 (0.88, 1.74) | 0.217 |
| Q3 | 0.91 (0.64, 1.28) | 0.573 | 0.99 (0.69, 1.43) | 0.984 | 0.96 (0.67, 1.4) | 0.845 | 0.98 (0.67, 1.41) | 0.912 |
| Q4 | 1.44 (1.15, 1.96) | 0.022 | 1.63 (1.17, 2.28) | 0.004 | 1.51 (1.14, 2.18) | 0.029 | 1.52 (1.05, 2.09) | 0.027 |
| *p* for trend | 0.039 | | 0.018 | | 0.042 | | 0.031 | |

Model 0: Non-adjusted.

Model 1: Sex, age, education, race, marital status, and PIR.

Model 2: Model 1 + BMI, smoking status, drinking habits, lymphocyte count, neutrophil count, hemoglobin, and physical activity.

Model 3: Model 2+ hypertension, diabetes, hyperlipidemia, CHD, CKD, depression, cancer, anti-hypertensive drug, anti-diabetic drug.

**Table S5b.** Association between MHR and all-cause mortality after exclusion of participants on anti-hyperlipidemic drug.

| **Variable** | **Model 0** | | **Model 1** | | **Model 2** | | **Model 3** | |
| --- | --- | --- | --- | --- | --- | --- | --- | --- |
|  | **HR (95%CI)** | ***p*-value** | **HR (95%CI)** | ***p*-value** | **HR (95%CI)** | ***p*-value** | **HR (95%CI)** | ***p*-value** |
| MHR continues | 2.61 (1.26, 5.43) | 0.011 | 2.81 (1.16, 5.81) | 0.022 | 2.86 (1.2, 5.82) | 0.018 | 3.18 (1.11, 7.08) | 0.031 |

Model 0: Non-adjusted.

Model 1: Sex, age, education, race, marital status, and PIR.

Model 2: Model 1 + BMI, smoking status, drinking habits, lymphocyte count, neutrophil count, hemoglobin, and physical activity.

Model 3: Model 2+ hypertension, diabetes, hyperlipidemia, CHD, CKD, depression, cancer, anti-hypertensive drug, anti-diabetic drug.
